# Editorial: Updates on Ocular Trauma

**DOI:** 10.3389/fmed.2022.906253

**Published:** 2022-04-28

**Authors:** Haoyu Chen, Vishal Jhanji, Rupesh Agrawal, Hua Yan

**Affiliations:** ^1^Joint Shantou International Eye Center, Shantou University & the Chinese University of Hong Kong, Shantou, China; ^2^University of Pittsburgh School of Medicine, Pittsburgh, PA, United States; ^3^National Healthcare Group Eye Institute, Tan Tock Seng Hospital, Singapore, Singapore; ^4^Department of Ophthalmology, Singapore Eye Research Institute, Singapore, Singapore; ^5^Lee Kong Chian School of Medicine, Singapore, Singapore; ^6^Duke National University of Singapore (NUS) Medical School, Singapore, Singapore; ^7^Department of Ophthalmology, Tianjin Medical University General Hospital, Tianjin, China

**Keywords:** ocular trauma, intraocular foreign body, chemical burn, traumatic macular hole, open globe injury, ophthalmic trauma registry

Ocular trauma is a significant cause of visual impairment worldwide. It is estimated that 19 million people worldwide have uniocular blindness from traumatic injury (1). Severe ocular trauma remains a challenge for physicians and may cause permanent blindness and even the loss of an eyeball. As ocular trauma refers exclusively to globe trauma, the use of the term “ophthalmic trauma” has been recommended to involve globe, adnexal, and orbital trauma. Recently, advances in research and data analytics have promoted our understanding and management of ocular trauma. Molecular biology of human tissues and animal models have revealed inflammation with unique molecular pathways in the pathogenesis of various types of ocular trauma (2). The application of *in vivo* imaging techniques, such as optical coherence tomography (3) and adaptive optics laser scanning ophthalmoscopy, has also helped us better understand the mechanism of damage to ocular tissues by external injuries. The advances of microinvasive vitrectomy, other surgical techniques, and novel surgical materials have improved the outcome of ocular trauma. The International Globe and Adnexal Trauma Epidemiology Study (IGATES) registry has furthered the research in ophthalmic trauma by facilitating global collaboration through a secure web-based platform for capturing both prospective and retrospective data by asking critical questions in patients with an eye injury (4). Novel tools such as the Ophthalmic Trauma Correlation Matrix (OTCM) have been proposed and evaluated for better management of patients with open globe injury (5). The Research Topic, *Updates on Ocular Trauma*, brings readers up-to-date laboratory, imaging, clinical, and epidemiological research on advances in ocular trauma.

An animal model would provide insight into the pathogenesis of ocular trauma and the development of potential treatment. Liu et al. established a new rabbit model of closed globe blast injury using a self-developed gas shock device. The equipment had a high-pressure air compression pump and could deliver different blast pressures of gas to the eyes. They found corneal edema, anterior chamber hyphema, lens opacity, vitreous hemorrhage, commotio retinae, and retinal ganglion cell damage in rabbit eyes with gas shock. Moreover, the severity of injury depended on the pressure. This rabbit model nicely replicates clinical closed globe injury.

Epidemiological studies can identify the prevalence and risk factors of ocular trauma, which would help to develop preventive strategies. Zhang et al. investigated the epidemiology of sports-related eye injuries among athletes. They found that 10.7% of athletes had a history of sports-related eye injury. Handball, water polo, and diving were the top types of sports that injured the eyes. Balls and teammates are both important sources of eye injury. An adnexa wound was the most common type of injury. About 11.9% of the eye injuries resulted in impaired visual acuity. The risk factors for eye injury included <18 years old, lower family income, and training > 4 h a day. Yu et al. reviewed the prevalence, spectrum, and management strategies of ocular trauma during the COVID-19 pandemic. They found that the prevalence of eye trauma decreased with a trend of delayed treatment during the COVID-19 pandemic. The possible mechanism may be irregular epidemic prevention and control measures, unprotected home activities, and unusual mental states. They discussed several strategies targeting these mechanisms for the prevention of eye injury during the COVID-19 pandemic.

Analysis of clinical spectrum and outcomes would benefit our understanding of ocular injury. Chen et al. analyzed 622 adult patients with light perception or no light perception after open globe injury. They found that 8.5% of the eyes received primary evisceration because of no possible anatomical reconstruction. There were 239 eyes that received vitreoretinal surgery. Over the 6-month follow-up, 21.9% of eyes were eviscerated, 24.0% atrophied, only 21.1% had some visual acuity, and 8.5% had vision better than 0.3. In Chen's other study of pediatric open globe injury, they found the most common injury type was penetration (89%). Scissors, knives, pens, and wood were common causes. Most (76.1%) of the injuries were in zone 1. After management, 32.2% had vision better than 0.3, and 2.7% were eviscerated. Su et al. reported a case with central retinal artery occlusion after a bee sting on the face, accompanied by hypersensitivity, hypercoagulable state, myocardial damage, and hepatic damage.

Surgical interventions are usually needed for severely injured eyes. Wang et al. reported allogeneic cultivated limbal epithelial cell sheet transplantation for severe symblepharon after chemical and thermal burns in 36 cases. Complete lysis was achieved after one surgery in 83.3% of cases and two surgeries in 16.7% without recurrence. Ma et al. compared the paths of giant intraocular foreign body (IOFB) removal in 73 cases. They found that eyes with IOFBs removed from the entrance wound path had a better visual outcome than those with a limbal path. The entrance wound path also allows for larger intraocular foreign body removal compared to the pars plana path. Chen et al. reported two novel suturing techniques to fix traumatic choroidal avulsion in 24 cases. Both techniques, trans-scleral mattress suturing and intraocular suturing, reattached the choroid well and improved both visual acuity and intraocular pressure. Zhou et al. conducted a systematic review and meta-analysis of vitrectomy vs. spontaneous closure of a traumatic macular hole. They found that the pooled macular hole closure rate was 0.37 in the observation group and 0.90 in the surgery group. The pooled rate of visual acuity improvement was 0.39 in the observation group and 0.72 in the surgery group. These articles provide evidence for clinicians to determine interventions for patients with ocular injury.

Studies on predicting outcomes would help us manage patients. Mai et al. reported that eyes with intraocular foreign body injury had generally delayed implicit time and reduced amplitude in all waves of five electroretinogram responses. The maximum change was found in oscillatory potential, which also had the strongest correlation with visual outcome. Their results would help select the appropriate ERG parameter and predict the visual outcome. Mohamed-Noriega et al. reported that the neutrophil to lymphocyte ratio and platelet to lymphocyte ratio were correlated with the visual outcome after surgical repair of open globe injuries in 197 cases. He et al. reported that the incidence of band keratopathy was 28% in a silicone oil tamponade for open globe injury. Silicone oil retention time ≥6 months and zone III injury were significant risk factors for band keratopathy.

In general, the articles on this Research Topic and current advancements in the field of ophthalmic trauma have provided updated information on the animal model, incidence, risk factors, clinical spectrum, precise diagnosis, intervention, and prognosis of a patient with an eye injury. There are some potential difficulties in the research of ocular trauma. There is a lack of large sample size high-quality datasets with long-time follow-up. It is not easy to conduct randomized control trials because of highly heterogenous clinical characteristics. Multicenter, even multinational collaboration is needed to collect large sample size, high-quality data and conduct randomized control trials to provide higher-level evidence for clinical decisions (4). The secure web-based global all-encompassing registries like IGATES, can provide real-time pooled data analysis that will not only advance our understanding in the field of ophthalmic trauma but also help us with predictive modeling and novel tools for evidence-based counseling of a trauma victim (4).

## Author Contributions

HC and HY conceived the idea. RA and VJ critically reviewed the manuscript and revised the article. All authors contributed to the article and approved the submitted version.

## Funding

This work was supported in part by the 2020 Li Ka Shing Foundation Cross-Disciplinary Research Grant (2020LKSFG14B) and Natural Science Foundation of Guangdong Province (2018A0303130306).

## Conflict of Interest

The authors declare that the research was conducted in the absence of any commercial or financial relationships that could be construed as a potential conflict of interest.

## Publisher's Note

All claims expressed in this article are solely those of the authors and do not necessarily represent those of their affiliated organizations, or those of the publisher, the editors and the reviewers. Any product that may be evaluated in this article, or claim that may be made by its manufacturer, is not guaranteed or endorsed by the publisher.
